# The relation between teaching-related self-efficacy and general job-related well-being – a cross-sectional study among young physicians

**DOI:** 10.3205/zma001777

**Published:** 2025-09-15

**Authors:** Benjamin Kiver, Pascal O. Berberat, Martin Gartmeier

**Affiliations:** 1 TUM MEC, TUM School of Medicine and Health, Technical University of Munich, Chair of Medical Didactics, Medical Teaching Development and Educational Research, Munich, Germany

**Keywords:** self-efficacy in teaching, emotional exhaustion, job satisfaction, job motivation, general job-related well-being, teaching experience

## Abstract

**Objective:**

The development of didactic skills plays a relatively subordinate role in medical training. However, teaching makes a central contribution to the training of young physicians and is an important part of the profession. In the present study, we therefore examine the question of how physicians’ teaching-related self-efficacy is related to aspects of their general job-related well-being. This construct is measured via the three components job satisfaction, job motivation and emotional exhaustion. From the results, we derive starting points for measures to increase the teaching-related self-efficacy of lecturers and the quality of teaching in clinical settings in the future.

**Method:**

Between 10/2016 and 09/2018, participants in university didactics training courses for medical lecturers at the Technical University of Munich (TUM) University Hospital were surveyed in writing. On this basis, we were able to analyze data from 293 participating physicians. We examined the assumed connections between teaching-related self-efficacy and emotional exhaustion, job satisfaction, and job motivation using Pearson correlations. For the relation to teaching experience, we calculated a Spearman correlation. We examined by *t*-test a possible difference in teaching-related self-efficacy between physicians who had received didactic training prior to our training vs. those who had not.

**Results:**

The study showed a statistically significant correlation between teaching-related self-efficacy and job satisfaction (*r*=0.138; *p*=0.020) as well as with job motivation (*r*=0.278; *p*<0.001). There was no statistically significant correlation with emotional exhaustion (*r*=-0.087; *p*=0.147) at work. Furthermore, teaching experience correlated positively with teaching-related self-efficacy (*ρ*=0.186; *p*=0.002) and physicians rated themselves significantly more self-efficacious in teaching if they had previously completed didactics training (*t*(282)=2.684, *p*=0.008).

**Conclusion:**

The teaching-related self-efficacy of physicians teaching at university correlated closely with the aspects of job satisfaction and job motivation, but not with emotional exhaustion. These findings offer starting points for interventional studies to investigate causal relationships that foster approaches to promote physicians’ teaching-related self-efficacy.

## Zusammenfassung

**Zielsetzung:**

In der ärztlichen Ausbildung spielt die Entwicklung didaktischer Kompetenzen eine untergeordnete Rolle. Jedoch leistet Lehre einen zentralen Beitrag in der Ausbildung junger Ärzt:innen und bildet für junge Lehrende einen wichtigen Bestandteil ihres Berufes. In der vorliegenden Studie untersuchen wir daher die Frage, wie die lehrbezogene Selbstwirksamkeit von Ärzt:innen mit Aspekten ihres allgemeinen arbeitsbezogenen Wohlbefindens zusammenhängt. Dieses Konstrukt wird dabei über die drei Komponenten Jobzufriedenheit, Jobmotivation und emotionale Erschöpfung erfasst. Aus den Ergebnissen leiten wir Ansatzpunkte für Maßnahmen ab, um zukünftig die lehrbezogene Selbstwirksamkeit von Dozent:innen und die Lehrqualität in Kliniken zu erhöhen.

**Methode:**

Im Zeitraum zwischen 10/2016 und 09/2018 wurden Teilnehmer:innen an hochschuldidaktischen Weiterbildungen für medizinische Dozent:innen am TUM Klinikum schriftlich befragt. Auf dieser Basis konnten wir Daten von 293 teilnehmenden Ärzt:innen auswerten. Wir untersuchten die angenommenen Zusammenhänge von lehrbezogener Selbstwirksamkeit mit der emotionalen Erschöpfung, Jobzufriedenheit und Jobmotivation mittels Pearson-Korrelationen. Für den Zusammenhang mit der Lehrerfahrung berechneten wir eine Spearman-Korrelation. Einen möglichen Unterschied der lehrbezogenen Selbstwirksamkeit zwischen Ärzt:innen mit einem unserer Schulung vorausgehenden vs. keinem vorausgehendem Didaktik Training prüften wir mittels eines *t*-Tests.

**Ergebnisse:**

In der Studie zeigte sich eine statistisch signifikante Korrelation zwischen lehrbezogener Selbstwirksamkeit und Jobzufriedenheit (*r*=0,138; *p*=0,020) sowie mit Jobmotivation (*r*=0,278; *p*<0,001). Kein statistisch signifikanter Zusammenhang zeigte sich mit emotionaler Erschöpfung (*r*=-0,087; *p*=0,147) im Beruf. Des Weiteren korrelierte Lehrerfahrung signifikant positiv mit der lehrbezogenen Selbstwirksamkeit (*ρ*=0,186; *p*=0,002) und Ärzt:innen schätzten sich signifikant selbstwirksamer in der Lehre ein, wenn sie in der Vergangenheit ein Didaktik-Training absolviert hatten (*t*(282)=2,684, *p*=0,008).

**Schlussfolgerung:**

Die lehrbezogene Selbstwirksamkeit in der Hochschullehre tätiger Ärzt:innen korrelierte eng mit den Aspekten Jobzufriedenheit und Jobmotivation, jedoch nicht mit emotionaler Erschöpfung. Diese Erkenntnisse eröffnen Ansatzpunkte für interventionelle Studien zur Untersuchung kausaler Zusammenhänge, um Kliniken Ansätze zur Förderung der lehrbezogenen Selbstwirksamkeit von Ärzt:innen aufzuzeigen.

## 1. Introduction

In addition to patient care, research and teaching are among the core responsibilities of physicians working at a university hospital. Given the limited financial and time resources, doing justice to all three areas of responsibility represents a particular challenge. Where research is concerned, there are structurally better opportunities to expand staff resources, e.g., by acquiring third-party funding or taking time off to conduct studies. By contrast, a high level of commitment to teaching is usually neither particularly encouraged nor rewarded. In consequence, physicians often lack the necessary time and incentives to set themselves ambitious didactic goals [1]. This relative neglect of teaching has negative effects, including on students, who often find the teaching they receive to be unsatisfactory [1]. Moreover, since the training of didactic skills does not play a role in medical studies[2], many physicians feel inadequately prepared when they are asked to design courses as part of their first employment at a university hospital [3]. However, since good teaching is a decisive factor in the quality of training for capable physicians, their didactic competence is of great importance for the entire medical profession. One question that has so far been neglected in this context is what role teaching competence plays in the job-related well-being of young physicians, who often find themselves thrust into the role of teachers, with little previous experience and medical didactic expertise. This might contribute to them feeling overwhelmed or out of place, and their job-related well-being could suffer as a result. 

**Figure 1:**
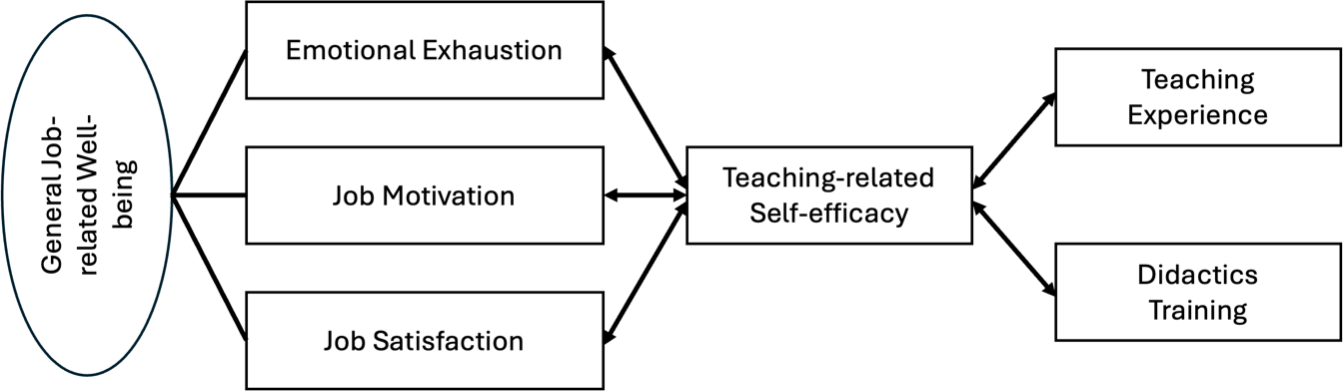
Hypothetical correlation between variables and structure of the construct of well-being

Our study builds on existing research on the cognitive characteristics of teachers, which from a theoretical perspective are more directly relevant to medical teaching [4]. Specifically, we shed light on a possible relationship between physicians’ teaching-related self-efficacy and aspects of their general job-related well-being, which we operationalize in the present study through the aspects of emotional exhaustion, job satisfaction, and job motivation. In addition, we examine the relation of teaching-related self-efficacy with teaching experience and with the completion of medical didactic training or further education in the past. The postulated associations are highly relevant, as higher job-related well-being itself is associated with positive job-related factors, such as performance or health [5], [6]. Therefore, a positive association between teaching-related self-efficacy and positive general work factors is evident. The results of our study contribute to understanding the importance of physicians’ didactic skills in general and in particular regarding the individual well-being of physicians. The theoretical concepts central to this article and their relation to each other are presented in more detail below (see figure 1 (Fig. 1)). All hypotheses were only formulated after data collection, rendering the analyses exploratory.

### 1.1. Teaching-related self-efficacy 

Self-efficacy refers to an individual’s confidence in their own resources for coping effectively with certain challenges and tasks. The concept was coined by Bandura [7] and is the subject of widespread and ongoing research today. According to Lent and Brown [8], self-efficacy lays the foundation for well-being, personal success, and motivation. In addition to global self-efficacy, it is assumed that domain- or task-specific self-efficacy, such as teaching-related self-efficacy, also exist [9]. Teaching-related self-efficacy refers to the assessment of one’s own abilities to design didactic measures that best support students in achieving specific learning goals [10], [11].

Klassen and Chiu [12] found a positive correlation between teachers’ self-efficacy regarding their own teaching and their job satisfaction. In addition, teachers with higher self-efficacy showed lower stress levels [13]. High teaching-related self-efficacy also appears to have a positive effect on learners: In a study of 103 primary school teachers and their 2,148 pupils, a positive correlation between teaching-related self-efficacy and academic performance was demonstrated [14]. These findings underpin the relevance of teachers’ own teaching-related self-efficacy, which has also previously been highlighted in the context of medical didactic research.

In the context of medical didactics, the focus of research to date has primarily been on the self-efficacy of medical students. Wu et al. [15] were able to show that higher intrinsic and extrinsic motivation is associated with increased self-efficacy. It has also been shown that self-efficacy is a variable critical to the learning process for medical students [16]. With regard to teaching-related self-efficacy in particular, Tipwong et al. found in a study of 227 physicians that teaching-related self-efficacy is a significant predictor of professional self-realization and is also negatively correlated to the risk of burnout [17]. In addition, a positive correlation between job satisfaction and teaching-related self-efficacy has been found for physicians [18]. Overall, it is clear that teaching-related self-efficacy is a relevant variable with regard to the quality of medical teaching. To date, however, there has been little research in the context of German university hospitals on the relationship between physicians’ teaching-related self-efficacy and intrapersonal characteristics. 

### 1.2. General job-related well-being

The focus of this study is on general job-related well-being, which we operationalize using three variables that are elucidated below. In our study, general job-related well-being is represented by the variables emotional exhaustion, job satisfaction, and job motivation [19]. 

#### 1.2.1. Emotional exhaustion 

The COVID-19 pandemic increased public awareness of the emotional exhaustion suffered by physicians and nursing staff [20]. Emotional exhaustion refers to a chronic state of physical and mental exhaustion caused by the continuous high or very high demands of work [21], [22] or study [23]. Emotional exhaustion plays a special role in medicine – though not yet in medical didactics – as this field is characterized by high social expectation and a high work intensity (at university hospitals not least due to teaching obligations). Doctors are a vulnerable group in terms of emotional exhaustion. This is confirmed by a Canadian study of 131 doctors, 80% of whom described symptoms of moderate to severe emotional exhaustion [24]. This is relevant because emotional exhaustion exacerbates the likelihood burnout [25], and is negatively associated with job performance [26]. In a study of 508 teachers, teaching-related self-efficacy was found to be the strongest negative predictor of emotional stress [27]. In a Thai study of 227 teaching physicians, teaching-related self-efficacy was shown to be a statistically significant negative predictor of burnout. Based on this, we assume the following hypothesis for the present study:

H1: Physicians’ teaching-related self-efficacy correlates negatively with their overall emotional exhaustion.

#### 1.2.2. Job satisfaction

Teaching-related self-efficacy also plays an important role in job satisfaction. Job satisfaction is a multidimensional psychological construct. It is defined as positive emotions that are triggered in an individual by their work or professional experiences [28], [29]. Job satisfaction is critical, not only in terms of job performance [30], but health [31]. The relation between teaching-related self-efficacy and job satisfaction has been widely researched and correlations have been confirmed in numerous studies for different occupational groups [32], [33], [34]. Buric and Moe [35], for example, found a significant relationship between teaching-related self-efficacy and job satisfaction in their study of 1214 teachers [18]. We build on this with the following hypothesis: 

H2: Physicians’ teaching-related self-efficacy of correlates positively with their job satisfaction.

#### 1.2.3. Job motivation

In order to create a working environment in which doctors can consistently perform at a high level, understanding their motivation is crucial [36], [37]. Job motivation correlates with health [38], while self-efficacy in turn is an important factor influencing job motivation [39], [40]. Demir [41] found in a study of teachers that an increase in their self-efficacy was associated with increased work motivation. However, the association of teaching-related self-efficacy with job motivation has not yet been confirmed in the field of medical didactics. We therefore put forward the following hypothesis:

H3: Physicians’ teaching-related self-efficacy correlates positively with their job motivation.

### 1.3. Teaching experience

Another potentially relevant factor influencing teaching-related self-efficacy is the extent of relevant experience in university teaching [42]. In a study by Gale et al. (2021), 67% of the participants surveyed cited positive teaching experience as a cause of increased teaching-related self-efficacy [43]. In their study of experienced and inexperienced teachers, Tschannen-Moran and Hoy [10] were able to show that experienced teachers rated themselves as significantly more self-efficacious. Applied to the clinical sector, we therefore assume the following hypothesis: 

H4: Physicians’ teaching experience correlates positively with their teaching-related self-efficacy.

### 1.4. Didactics training

A further aim of this study is to investigate whether the completion of higher education didactics training is associated with a higher teaching-related self-efficacy assessment by physicians themselves. Typical elements of such training courses are in-depth discussions of teaching-learning theories and modern approaches to higher education didactics, structured and focused testing of current teaching-learning methods, and supervision of one’s own teaching activities with feedback. These elements can help to promote didactic skills. We therefore see university didactics training as a useful prerequisite for increasing teaching-related self-efficacy assessment.

The benefits of interpersonal skills training (and we consider didactic action as such) are well documented, including for the medical profession. Ammentorp et al. [44] found that clinical and nursing staff were able to increase their self-efficacy in terms of communication by an average of 37% with five days of communication training. In a meta-analysis by Mata et al. [45], the positive effect of various communication training courses on the self-efficacy of physicians was confirmed. The benefits of specialized didactics training for university teaching have also been confirmed in numerous studies. In his study, Tzivinikou [46] showed that didactics training for teachers significantly increased their teaching-related self-efficacy. Based on these correlations, we postulate the following hypothesis:

H5: Physicians who have completed didactic training differ significantly in their assessment of their own teaching-related self-efficacy from study participants who have not completed didactic training.

## 2. Methods

To investigate the hypotheses formulated above, we use data from the scientific monitoring of a university didactic training program for medical lecturers conducted between 10/2016 and 09/2018 using standardized questionnaires. All new employees with teaching duties at the TUM University Hospital are required to complete a lecturer training course within their first year. The lecturer training takes place in the form of lectures, discussions, and group work on various didactic topics and is supervised by members of the chair of medical didactics. The participants are therefore predominantly young professionals at the start of their careers, although all employees with teaching duties are free to particpate in the training on a voluntary basis. 

As part of the training, we informed all participants about the study and asked them to complete the questionnaire. Participation in the study was voluntary; if participants decided not to take part, this had no negative consequences for them. Our study was a cross-sectional. The underlying data was collected at 13 points in time as part of the presence-based training. In addition, an e-mail was sent out from the TUM University Hospital to recruit additional doctors online. It is therefore not possible to state the maximum number of participants that could be recruited or the response rate. A total of 315 people took part in the study, with data from 293 trial participants included in the analyses. 22 people were excluded as they were not physicians. 

### 2.1. Instruments of measure

Established questionnaires on perceived self-efficacy in teaching, emotional exhaustion, job satisfaction, and job motivation were used to record the variables in the focus of the study (see table 1 (Tab. 1)). A four-point Likert scale ranging from “strongly disagree” to “strongly agree” was used for all instruments of measure. Perceived self-efficacy in teaching was assessed using items after Pfitzner et al. [47], with some adaptations. The scale showed good reliability with a Cronbach’s alpha of 0.75. To measure job motivation, we used the eight-item scale derived from Bakker et al. [48], which also showed good reliability with a Cronbach’s alpha of 0.84. The four items on emotional exhaustion were adapted from Maslach [49]; the scale had a Cronbach’s alpha of 0.82. The job satisfaction scale comprised four items and was developed by Hackman and Oldham [50]; it had a Cronbach’s alpha of 0.85. If all constructs operationalized here as partial aspects of general job-related well-being (i.e., the items of the emotional exhaustion (inverted), job satisfaction and job motivation scales) are combined, the scale has a Cronbach’s alpha of 0.89.

Participants were asked about their teaching experience in four categories: up to one year, 2-5 years, >5-10 years, and over 10 years of teaching experience. Participation in teaching didactics training of any kind prior to our training was also recorded with a dichotomous yes/no item.

### 2.2. Statistical analyses

The analyses were based on a significance level of p=0.05. Pearson correlations were calculated to test the first three hypotheses; the fourth hypothesis was tested using a Spearman correlation. A t-test was used to test the fifth hypothesis. All analyses were carried out using IBM SPSS 28 software. No power analysis was performed in advance. To analyze the missing items, we performed a data missing completely at random test (MCAR test) after Little. This resulted in a χ^2^ value of 489.882 with df=465 and a *p*=0.205. Since the *p*>0.05, we cannot reject the null hypothesis. This indicates that the missing values are missing completely at random. Finally, the missing values were eliminated by mean imputation. In the statistical tests, pairwise case exclusion was used when all items of a scale of one of the correlating variables were missing. 

### 2.3. Ethical approval

Participation in the study was voluntary and anonymous. Ethics approval for the study was obtained from the Ethics Committee of TUM University Hospital (Klinikum Rechts der Isar) (reference 487/19 S-KK).

## 3. Results

### 3.1. Sample description

The majority of participants (94.5%) were employees at the TUM University Hospital (Rechts der Isar). The participants were predominantly male, between 25 and 39 years old and had already obtained a doctorate. The majority of participants also had less than one year of teaching experience and had not previously completed any didactics training. Our sample was a conveniance sample with 315 participants. The detailed characterization of the sample, including the missing values, can be found in table 2 (Tab. 2).

### 3.2. Emotional exhaustion, job satisfaction, job motivation

On average, the doctors surveyed in the study were not especially emotionally exhausted (*M*=2.93, *SD*=0.70, *N*=288; recoded scale with maximum (4=not emotionally exhausted at all). Nevertheless, 61 people (21%) still reported themselves emotionally burdened to a relevant extent (48 people over 1 standard deviation below the mean, 13 people over 2 standard deviations below the mean). Contrary to the postulate in hypothesis 1, there was no significant correlation (*r*=-0.087; *p*=0.147; *N*=281) between the teaching-related self-efficacy of physicians and their emotional exhaustion at work. The questionnaire showed that the doctors surveyed were satisfied with their profession (*M*=3.05, *SD*=0.80) and motivated in their work (*M*=2.89, *SD*=0.54). Higher teaching-related self-efficacy among physicians correlated positively with their job satisfaction (*r*=0.138; *p*=0.020; *N*=283), and it also correlated significantly positively with the respondents' job motivation (*r*=0.278; *p*<0.001; *N*=278). 

### 3.3. The role of teaching experience and skills training 

With regard to the extent of teaching experience, there were differences in the various age categories. While physicians with up to one year of teaching experience rated their self-efficacy in teaching on average at *M*=2.81, *SD*=0.34, physicians with 2-5 years (*M*=2.93, *SD*=0.36), 5-10 years (*M*=2.98, *SD*=0.34) and over 10 years (*M*=2.91, *SD*=0.22) of teaching experience rated their self-efficacy higher. An analysis of variance of the individual response categories of teaching experience and teaching-related self-efficacy as the dependent variable produced a significant result (*F*(3, 277)=4.003, *p*=0.008). A post-hoc Tukey HSD test revealed a significant difference in teaching-related self-efficacy between the group with up to one year of teaching experience and the group with 5 to 10 years of teaching experience. As postulated in Hypothesis 4, teaching experience showed a significant correlation (*ρ*=0.186; *p*=0.002; *N*=281) with the teaching-related self-efficacy of physicians.

The majority of physicians in our sample had not yet completed any didactics training (204 vs. 78 people). Regardless of whether they had completed such training, the doctors in our study tended to rate themselves highly in terms of their own self-efficacy in teaching; nevertheless, there was a difference in favor of those doctors who had already completed didactics training. They rated themselves significantly more self-efficacious (*t*(282)=2.684, *p*=0.008) in teaching (*M*=2.96, *SD*=0.32) than the comparison group who had not previously attended such training (*M*=2.84, *SD*=0.35).

Overall, the significant results in our study showed small to medium effect sizes (see table 3 (Tab. 3)).

## 4. Discussion

Our study results suggest that experience in the teaching role is associated with aspects of physicians’ job-related well-being. We were able to demonstrate corresponding correlations of this role with job satisfaction and job motivation, but not with emotional exhaustion. Furthermore, we found that teaching experience plays an important role in physicians’ teaching-related self-efficacy. We also found higher teaching-related self-efficacy scores for the group of physicians who had already completed didactics training compared to the group without previous training. 

Our first hypothesis could not be confirmed: Teaching-related self-efficacy did not correlate significantly with physicians’ emotional exhaustion. There could be several reasons for this, including the fact that emotional exhaustion is multifactorial. We were thus unable to show the influence of teaching-related self-efficacy separately in our study. In contrast to the consideration of job motivation and job satisfaction, private life also represents a stronger influencing factor here. Klusmann and Aldrup [51], for example, found in their study that both positive and negative private experiences can have an impact on the emotional exhaustion of teachers. In subsequent studies, it would be useful to introduce a more holistic measure of emotional exhaustion as a control variable. At the same time, it is debatable whether it is fundamentally expedient to evaluate the emotional exhaustion scale as an indicator variable for general job-related well-being in future studies, even though the three aspects had a Cronbach’s alpha of a=0.89. For reasons of consistency, a reduction to four values was chosen for all scales. Applying the original number of values might have allowed a more differentiated picture.

Our results further confirm our assumption of a correlation between teaching-related self-efficacy and job motivation and job satisfaction. It seems logical that those who see themselves as competent and self-efficacious in their work are also more satisfied and motivated in their jobs. Job satisfaction and job motivation are dependent on many external and individual factors outside the field of teaching. Job satisfaction, for example, correlates with the “sense of belonging to the hospital” and emotional well-being [52]. In other studies, job motivation is positively associated with work-life balance [53]. A positive influence on teaching-related self-efficacy appears to be easier to achieve through targeted training measures than, for example, the manipulation of variables such as a sense of belonging or work-life balance. 

Like Prieto and Altmaier [54], we were able to demonstrate a positive correlation between physicians’ teaching experience and their teaching-related self-efficacy. Those who had spent a longer period of time in teaching had a greater chance of having had lasting experience of success, which is an important source of teaching-related self-efficacy [43].

In our study, the groups of participants who had previously completed non-subject-specific university didactics training differed significantly in their teaching-related self-efficacy from those who had not yet completed any training at the time of the study [45], [55]. Future research could clarify these findings through interventional study designs, e.g., with regard to the question of which training content or which didactic methods are most conducive to teaching-related self-efficacy. 

By offering subject-specific didactics training, medical faculties and university hospitals can directly promote the teaching-related self-efficacy of their employees and thus potentially have a positive influence on their job-related well-being. If necessary, initial course offerings could also be made during medical studies, e.g., to prepare medical students for didactic activities as instructors or peer teachers. In addition, teaching in the clinic should be prioritized more strongly, in the form of extensive didactics training before the first teaching session, while at the same time relieving students of their daily tasks.

Various limitations of the present study should be mentioned: Our sample can be characterized as an opportunistic sample collected monocentrically. However, since local conditions differ between different university hospitals and medical faculties, its generalizability is limited. Furthermore, in the theoretical derivation of our hypotheses, we argued with causal justifications, for example with regard to the role of self-efficacy in teaching as an influencing factor on job-related well-being However, due to the cross-sectional design of our predominantly correlative study, we cannot draw any causal conclusions. In addition, we were unable to capture some potentially relevant influencing factors in our study, such as physicians’ intrapersonal dispositions relevant to teaching or the extent of teaching actually undertaken. Furthermore, the physicians in our sample with more teaching experience were more likely to have previously completed medical didactic training. The correlations themselves showed only small to medium effects (see table 3 (Tab. 3)), but these are large constructs that affect the entire profession and the results should be interpreted in this light. Furthermore, with regard to the calculated analysis of variance, it should be noted that when grouping the participating lecturers with regard to their teaching experience, their age could not also be taken into account as a potentially independently relevant variable. Finally, when comparing individuals with and without previous didactics training, it was not specified which type of didactics training the respondents had completed. This could also be associated with distortion effects.

## 5. Conclusion

In this study, we were able to show positive correlations between physicians’ teaching-related self-efficacy and their job satisfaction and job motivation. In addition, teaching-related self-efficacy correlated positively with teaching experience and showed higher levels among physicians who had previously completed didactic training. These findings make it clear that an environment can be created at medical faculties that promotes teaching-related self-efficacy, e.g., by offering low-threshold, accessible but mandatory didactics training. In addition, young doctors at university hospitals should be involved in teaching at an early stage so that they can gain their first teaching experience and acquire didactic skills in parallel with medical and technical skills. 

## Authors’ ORCIDs

- Benjamin Kiver: [0009-0005-1053-0229]
- Pascal O. Berberat: [0000-0001-5022-5265]
- Martin Gartmeier: [0000-0002-5025-0003]

## Competing interests

The authors declare that they have no competing interests. 

## Figures and Tables

**Table 1:**
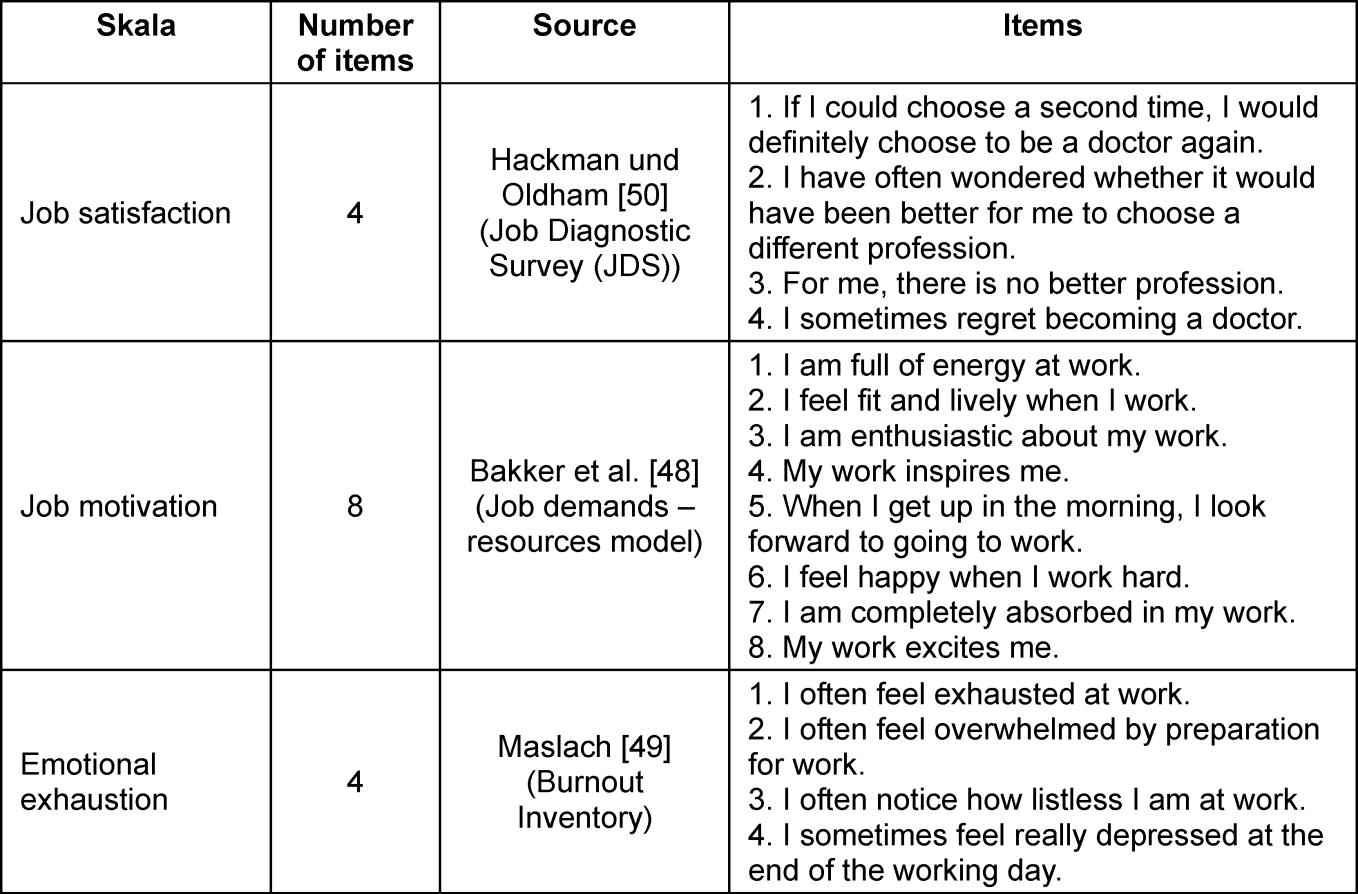
Overview of scales

**Table 2:**
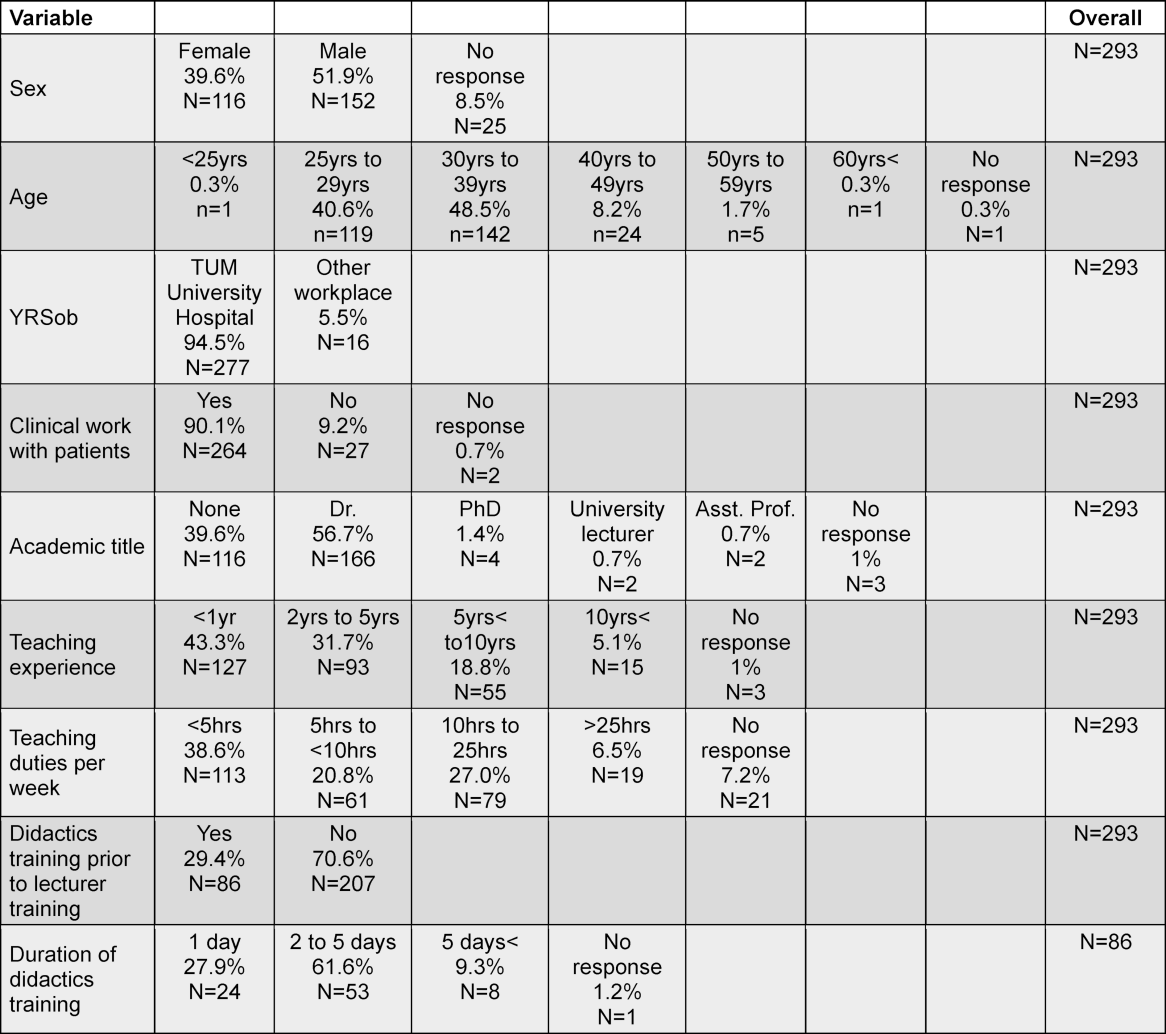
Sample description

**Table 3:**
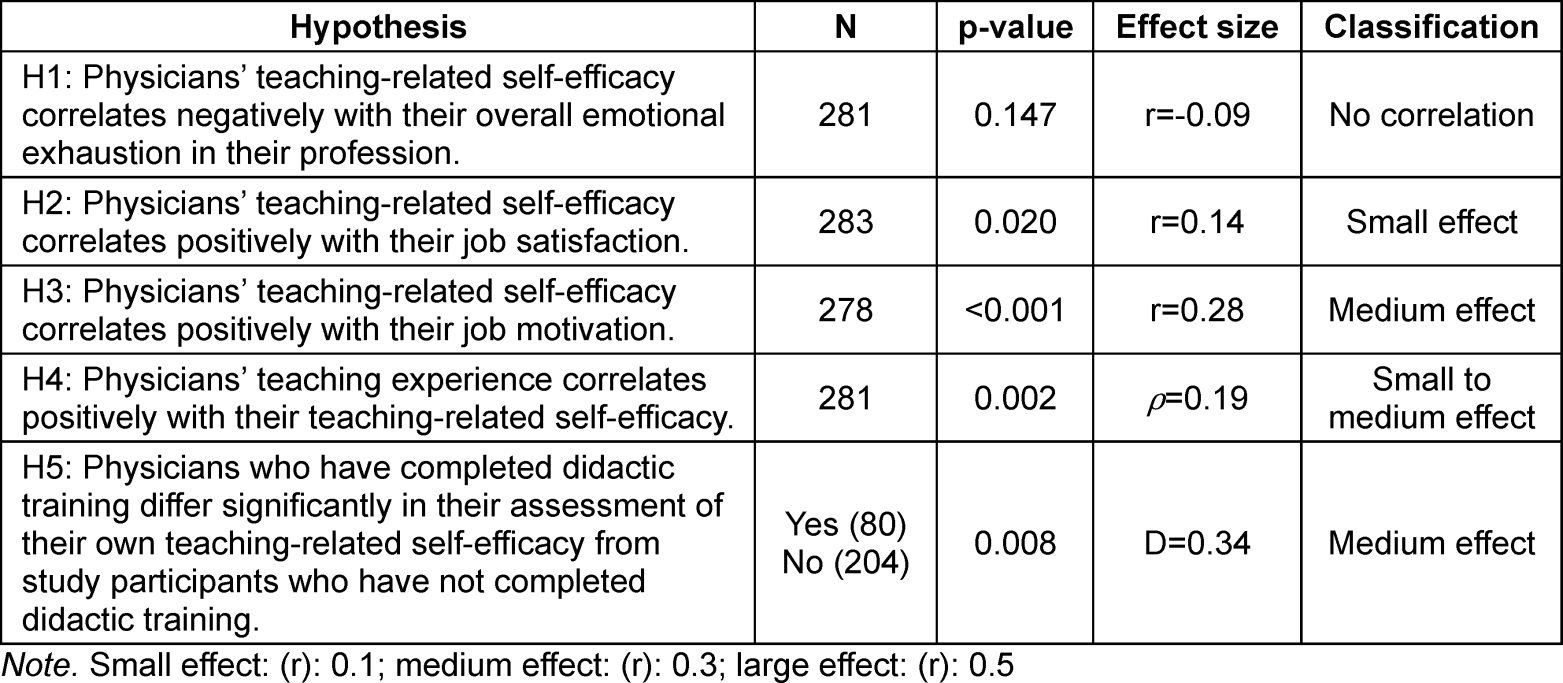
Overview of study results with effect sizes
